# Evaluation of Potentially Toxic Trace Metals and Associated Health Risk Assessment in Buffalo Milk

**DOI:** 10.3390/ijerph192214678

**Published:** 2022-11-09

**Authors:** Aqsa Rafiq, Munir H. Shah, Mohamed Mohany, Adnan Ahmad Tahir, Mohamed Farouk Elsadek, Muhammad Abdul Qayyum, Arshad Mehmood Abbasi

**Affiliations:** 1 Department of Environmental Sciences, COMSATS University Islamabad, Abbottabad 22060, Pakistan; 2 Department of Chemistry, Quaid-i-Azam University, Islamabad 45320, Pakistan; 3 Department of Pharmacology and Toxicology, College of Pharmacy, King Saud University, Riyadh 11451, Saudi Arabia; 4 Department of Community Health Sciences, College of Applied Medical Sciences, King Saud University, P.O. Box 10219, Riyadh 11433, Saudi Arabia; 5 Department of Chemistry, Division of Science & Technology, University of Education, Lahore 54770, Pakistan; 6 University of Gastronomic Sciences, Piazza Vittorio Emanuele II 9, 12042 Pollenzo, Italy

**Keywords:** buffalo milk, potentially toxic metals, statistical analysis, health risk, cancer risk

## Abstract

The contamination of toxic trace metals in the food chain is one of the major threats to human health. Milk is part of a balanced diet, which is essential for proper growth, but the ingestion of contaminated milk may cause chronic health disorders. The present study is focused on the assessment of contamination of toxic trace metals in buffalo milk and the associated health risks to the consumers of Abbottabad, Pakistan. Standard analytical methods were employed to quantify the metal contents in the milk samples collected from various shops and homes in the months from June 2021 to October 2021. Health risk assessment was accomplished by computing estimated daily intake (EDI), health risk index (HRI), target hazard quotient (THQ), hazard index (HI), and target cancer risk (TCR). On a comparative basis, the mean concentration of Cr was found to be highest in both shop and home milk samples (101.3 ± 45.33 and 54.11 ± 24.20 mg/L, respectively), followed by Pb, Zn, Ni, and Cd levels. In buffalo milk collected from homes, the highest concentration of the metals was found in October, followed by July, September, June, and August. In shop milk, the increasing trend of metal contents was July > October > September > June > August. Significantly strong positive relationships were noted between the metal concentrations in the milk samples. Multivariate cluster analysis and principal component analysis exhibited significant anthropogenic contributions of the metals in buffalo milk. Mostly, the EDI and HRI values were exceeding the recommended limits; however, THQ, HI, and TCR showed that the intake of these metals through milk consumption was within the safe limit and thus revealed no significant carcinogenic or non-carcinogenic risks to the consumers. It is high time to ensure the continuous monitoring of organic/inorganic toxins in the milk and concerned authorities should take strict measures to control the contamination of milk and other food products.

## 1. Introduction

Elevated concentrations of toxic elements in air, soil, water, and the food chain are considered as a major concern globally [1]. Anthropogenic activities resulted in the enrichment of potentially toxic metals in geological and biological systems [2] and pose a major hazard to human health [3]. Exposure to the toxic metals in humans is induced by the inhalation of airborne particles, ingesting contaminated dirt, or skin absorption [4]. These metals also accumulate in flora, fauna, and ultimately in the food chain [5]. It has been estimated that ≈90% of human exposure to most of the toxic metals is due to the intake of contaminated food, which is responsible for 30% of all human malignancies worldwide [6]. Various trace metals can accumulate in the food chain and the human body because of their higher absorption and poor extraction rates as well as long half-lives [7]. 

Some trace metals, such as, Fe, Zn, and Cu, are required in humans in minute quantities; thus, they are essential because of their nutritional and medicinal value [8,9]. However, beyond threshold levels, these trace metals are harmful to the living organisms and specifically cause various health problems in humans [10,11]. Some other trace metals, such as, As, Cd, Cr, Hg and Pb metals, have no known bio-relevance in human physiology or biochemistry, and their exposure can be hazardous even at very low concentrations [8]. Except for those that are bio-important, dietary consumption must be kept below the regulation limits because elevated levels of these metals can induce toxicity, which leads to physical problems and even death [12]. Even in small amounts, the frequent intake of toxic metals may cause serious impacts on human health, especially in infants and children; thus, they are of significant concern [13,14]. Exposure to various metal contents may cause morphological dysfunctions [15], mental retardation [16], disruption of the central nervous system, liver, prostate, and kidney disorders, hypertension, ulcers, cancers [1,8,17], asthma, osteoporosis, hormonal disturbances, gastrointestinal complications, immune system deterioration, and infertility [6,18].

Milk is an ideal food in terms of nutritional composition and offers instant energy due to its ease of digestion and absorption [19,20]. It is a good source of proteins, vitamins [21], calcium, amino acids, fatty acids, and bioactive ingredients, which are vital for the growth and maintenance of human health, providing strength to bones and teeth, as well as for preventing hypertension [22,23]. However, in recent years, milk contamination has been identified as one of the major public health risk factors [24]. Milk may contain inorganic/organic contaminants and more than twenty xenobiotic compounds; trace metals, mycotoxins, dioxins, and other pollutants have been reported in it [22,25].

It is well established that rapid industrialization and urbanization have resulted in increased levels of the toxic metals that ultimately find their ways into the milk and dairy products through various routes [26], such as through contaminated feed of cattle, poor quality drinking water, sewage runoff, extensive use of pesticides and fungicides, and industrial effluents near livestock feeding areas [27,28]. For instance, several workers including Lutfullah et al. [29] from Pakistan, Rezaei et al. [30] from Iran, Abdel Khalek et al. [31] from Egypt, and Ogabiela et al. [32] from Nigeria reported various toxic trace metals in milk, which were mostly above the permissible limits. 

Pakistan is the 4th largest milk producer in the world. However, ≅4% of milk in Pakistan is refined, and the remaining 96% is consumed as fresh milk, which is mostly supplied by milkmen recognized as “Doodhies” [33]. On a small scale, ≅80% of milk is produced in rural areas, followed by 20% in peri-urban and urban areas that constituting 15% and 5%, respectively. Average household holdings of cattle/buffaloes are three to four, and there are four to five sheep/goats per family. Livestock shares constitute more than 60% of the agriculture sector of Pakistan, and the country had processed more than 63 million tons of milk in 2020–2021, which accounts for 11.5% of gross domestic products (GDP). It is estimated that 40% of the livelihoods of 8 million families in Pakistan depend on milk and dairy products from cows and buffalos [34]. According to FAO/WHO [35], in Pakistan, cow milk contributes 83% of the total milk production followed by buffalo milk and goat milk (14% and 2%, respectively). In 2020–2021, per capita milk availability was about 172 L/annum. Punjab province of Pakistan has the largest population of buffaloes accounting for 65% of the total livestock, Sindh (26%), Khyber Pakhtunkhwa (7%), and Baluchistan (1.3%). Punjab and Sindh provinces of Pakistan produce 25.62 and 9.35 million liters of milk per year, respectively, while KP produces 4.88 million liters, and Baluchistan produces 0.81 million liters per year [34].

Buffalo milk is one of the most consumed types of the milk both in raw and pasteurized products in Pakistan. Like other parts of the country, in Abbottabad, city milk is supplied from shops as well as from home-based dairy animals owned by the local inhabitants. Fresh buffalo milk sold at shops is usually a mixture of milk supplied from different sources i.e., by home-based cattle owners, and transported from dairy forms in the neighboring areas of Punjab and Khyber-Pakhtunkhwa provinces. Therefore, it was hypothesized that buffalo milk available at shops contains more HMs compared to home-based milk from dairy animals. However, the concentration of various potentially toxic metals in buffalo milk consumed by the inhabitants of Abbottabad and its associated health risks to the consumers is not well explored yet. In this context, the present study was conducted with the aim (i) to quantify potentially toxic metals in the buffalo milk collected from shops and homes, and (ii) to assess the health risks associated with potentially toxic metal contents in buffalo milk to the consumers.

## 2. Materials and Methods

### 2.1. Sampling and Digestion 

**Figure 1:**
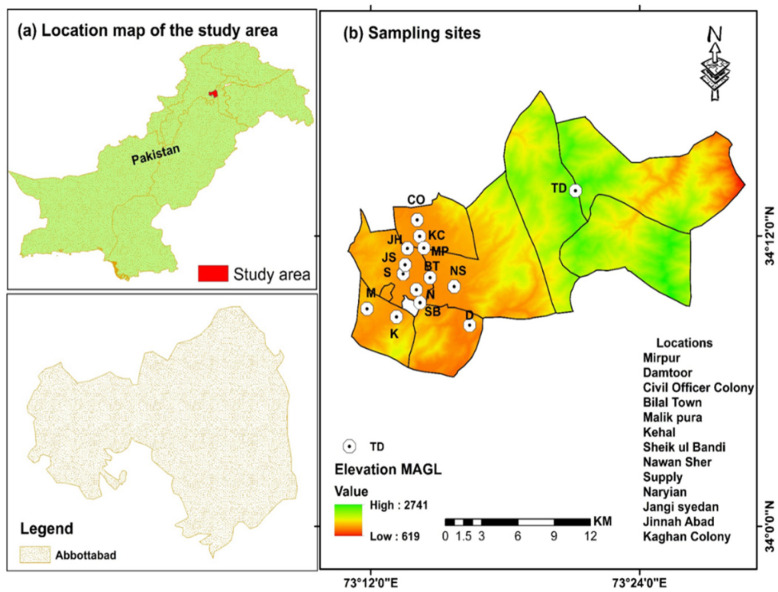
Map showing different sampling sites included in the present study. (**a**) indicates location map of the study area in Pakistan (**b**) showing sampling sites.

In the present study, we compared HMs in milk collected directly from homes where local people own dairy animals, and from shops where milk comes from different areas. Month-wise sampling was conducted from June 2021 to October 2021 following the method as explained earlier [36]. Fresh samples of buffalo milk (≈50 mL from each site) were collected randomly from shops and homes located at of thirteen different sites within the territory of Abbottabad city (Figure 1). From each site, 3–5 samples were collected (from homes and shops) and pooled to make a composite sample in one bottle. In each month, 78 composite samples in triplicate [(13 + 13) × 3] = 78) were collected from homes (13 sites) and shops (13 sites), and kept in clean, sterilized, and labeled glass bottles for further processing. The total number of samples used to quantify metals for the monitoring period from June to October was ≅390. 

Milk samples were digested following the method as described earlier [37], and all precautionary measures were taken. Briefly, 5.0 mL of each sample was accurately measured using a glass pipette and added into labeled conical flasks. Then, 10 mL HNO_3_ (65%), was added to each sample, and all samples were kept at room temperature overnight. The next day, each sample was heated on a hot plate in a fume hood at 100 °C for one hour. After cooling to room temperature, 5 mL HCIO_4_ (70%) was added to each sample and heated again at 120 °C, until a clear solution was obtained. After complete digestion, the samples were cooled to room temperature and diluted up to 50 mL with distilled water in cleaned and labeled volumetric flasks. One blank (the digestion mixture without sample) was also prepared and digested under the same conditions with a batch of 8 samples. The digested samples and blanks were kept at 4 °C before further analysis.

### 2.2. Metal Analysis

Five potentially toxic metals viz. Cd, Cr, Ni, Pb, and Zn were quantified in the digested samples of buffalo milk using an Atomic Absorption Spectrophotometer (Perkin Elmer-Waltham, MA, USA, S# 8015050702), following the procedure as explained earlier by Ismail et al. [18], under optimum analytical conditions as mentioned in Table S1 (Supplementary Materials). Stock solutions (1000 mg/L) of each metal were prepared using double-distilled water and used for preparing the working standards of different concentrations. For quality assurance, certified/standard reference material (NIST SRM 1515) was also analyzed under similar conditions, and it depicted an excellent recovery (98–100%). In addition, about 10% of the samples were used for inter-laboratory analysis, and variation in the results was less than ± 3.0%. 

### 2.3. Statistical Analysis

Data were analyzed using Microsoft Excel and presented as mean ± SD for triplicates. Correlation coefficient matrix, principal component analysis, and cluster analysis were also conducted using SPSS 13.0 version (SPSS Inc., Chicago, IL, USA).

### 2.4. Health Risk Assessment

Health risk assessment of the trace metals due to the consumption of fresh buffalo milk was evaluated by calculating different parameters/indices including health risk index, target hazard quotient, the health index, and target cancer risk. The daily intake of metals (DIM) was estimated following the method as reported previously [38,39]. Ingestion rates of buffalo milk in adults and children (0.150 and 0.330 kg/capita/day, respectively) were taken from previous reports [18]. The average body weights for adults and children (70 and 20 kg, respectively) were used as reported earlier [40]. Daily intake of the metals was calculated using the following formula:

(1) DIM=Cm×Ir/Bw

where Cm is the measured levels of trace metals in milk samples, Ir represents the ingestion rate of milk and Bw is the body weight of consumers. 

Health risk due to the metals was determined using the “health risk index (HRI)” as explained earlier by Cui et al. [41]. HRI is a proportion of daily intake and oral reference dose of a specific metal; HRI values of the metals less than unity (1.00) are considered safe for consumers, and more than unity is considered hazardous [42]. In buffalo milk samples, HRI was calculated using the formula:

(2) HRI=(Cm×Ir)/(RfD×Bw)

where Cm is the concentration of trace metals in milk samples, Ir is the average daily intake/ingestion rate of milk, RfD represents oral reference doses of the metals and Bw indicates the body weight of consumers. Oral reference doses of Cd, Cr, Ni, Pb, and Zn (0.001, 1.500, 0.020, 0.003, and 0.300 µg/g, respectively) were reported previously [43,44,45]. 

The target hazard quotient (THQ) and hazard index (HI) were calculated to assess non-carcinogenic risks to the consumers due to intake of the metals through consumption of buffalo milk. As reported by the USEPA [45], the computed values of THQ and HI less than one (<1) indicate that consumers do not have any significant health hazards due to intake of the metals, but THQ and HI values >1 reveal potential health risks to the consumers. The THQ in buffalo milk was estimated using the following formula, as reported earlier [46].

(3) $$\mathrm{HQ}=\left(\mathrm{Cm}\times \mathrm{Ir}\times 10^{-3}\times \mathrm{CtP}\times \mathrm{ElPtot}\right)/\mathrm{RfD}\times \mathrm{BW}\times \mathrm{AnCt})$$

where Cm indicates measured levels of the metals, Ir is the ingestion rate of milk, CtP is contact time-period (365 days/year), ElPtot represents total exposure length period (30 years), Bw is body weight of consumers, and AnCt designates average non-carcinogenic time: “ElPtot was taken 24 h a day, 7 days/week” [40].

Hazard index (HI) was calculated by adding the THQs estimated for all metals using the following formula as explained previously USEPA [45].

HI = THQ₁ + THQ₂+……. + THQ1-n(4)

where “THQ1-n = Target hazard quotients for 1-number of metals”. 

The cancer risk due to intake of the toxic metals was estimated by target cancer risk (TCR), which is a commonly used tool to determine the carcinogen risk assessment for contaminated food materials [45]. The TCR values ranging from 10^−6^ to 10^−4^ are considered safe and indicate no significant cancer risk, but beyond this limit, it is considered as significantly carcinogenic for the consumers [47]. The TCR in buffalo milk was calculated using the following equation:

(5) $$\mathrm{TCR}=\left(\mathrm{Cm}\times 1\times 10^{-3}\times \mathrm{CSF}\times \mathrm{Er}\times \mathrm{TEL}\right)/\left(\mathrm{Bw}\times \mathrm{ATp}\right)$$

where Cm is the concentration of metals in buffalo milk, CSF represents the oral cancer slope factor for “Cd, Cr, and Pb (6.100, 0.500, and 0.0085 mg/kg/day, respectively)”, Er is the exposure rate (days/year), TEL is the total exposure length (70 years), ATp is the average time for carcinogens, and Bw is the body weight of adults and children. 

## 3. Results and Discussion 

### 3.1. Distribution of the Metals in Buffalo Milk 

Measured levels of the potentially toxic metals in buffalo milk samples collected from homes and shops of different localities from June 2021 to October 2021 are presented in Table 1. In June, the highest concentrations of Cd, Cr, Ni, Pb, and Zn metals were determined in samples collected from homes located at Jangi Syedan, Malik Pura, Mirpur, Jinnah Abad, and Kaghan Colony. However, in milk samples collected from shops during the same month, Cd was maximum in Kehal, Cr was maximum in Supply, Ni was maximum in Mirpur, Pb was maximum in Jinnah Abad, and Zn concentration was highest in Kaghan colony. The milk samples collected from different homes of Jinnah Abad, Mirpur, Kehal, Nawan Sher, and Damtor during July exhibited the highest levels of Cd, Cr, Ni, Pb, and Zn, respectively. Likewise, in the same month, milk samples collected from shops of Sheik ul Bandi, Mirpur, Supply, and Malik Pura contained maximum contents of the studied metals. Measured levels of Cd were highest in the milk samples collected in August from homes and shops of Malik Pura, Kaghan colony, and Sheik ul Bandi. The measured level of Cr was highest at Bilal Town (in home samples) and Milak Pura (in shop samples). The concentration of Ni was below the detection limits in home and shop samples collected from all locations during August. Nevertheless, the measured levels of Pb and Zn were maximum in shop samples collected from Supply and Bilal Town, but in home samples, Pb was below the detection limit, while Zn was maximum in Mirpur. In September, Cd was below the detection limit in home and shop samples. However, Cr and Zn levels were highest in both types of the milk samples collected from Kaghan Colony. The measured concentration of Ni was highest in Jangi Syedan (in home milk) and Jinnah Abad (in shop milk) collected in September. Relatively, measured levels of Pb were highest in Bilal Town (in home milk) and Malik Pura (in shop milk). In October, Cr and Pb concentrations were maximum in home and shop samples collected from Malik Pura and Jinnah Abad sites, respectively. The estimated level of Cd was maximum in Nawan Sher (in home milk) and Nariyan (in shop milk), Ni was maximum in Jinnah Abad (in home milk) and Kaghan Colony (in shop milk), whereas Zn was highest in Kehal and Nawan Sher in home and shop milk samples, respectively (Table 1). 

**Figure 2:**
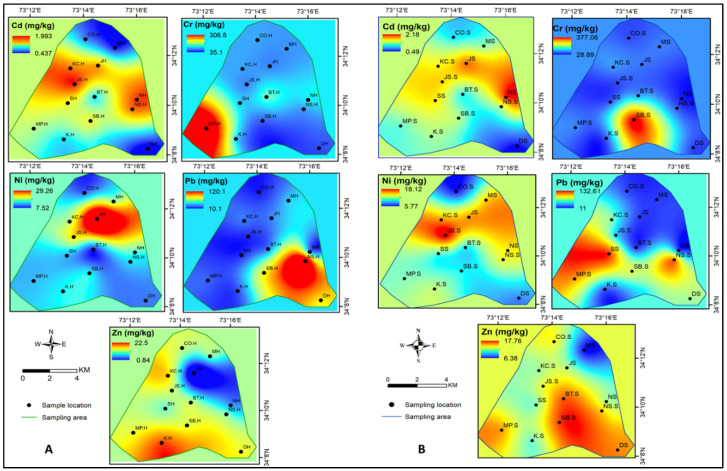
Spatial distribution of HMs qualified in buffalo milk collected from different locations (**A**) homes, (**B**) shops.

Spatial distributions of the trace metals in buffalo milk collected from homes and shops of various locations are illustrated in Figure 2A,B. Measured levels of Cd were comparatively higher in the milk samples collected from homes located in Jangi Syedan, Kaghan Colony, Nawan Sher, and Nariyan (Figure 2A). Likewise, the concentration of Cr was maximum in Malik Pura, while Ni was highest in Jinnah Abad, followed by Jangi Syedan, Kaghan Colony, and Mirpur areas. Highest concentration of Pb was found in the milk samples collected from different homes of Nawan Sher, Sheik ul Bandi, and Damtor, while Zn was maximum in Kehal. In the case of milk samples collected from shops (Figure 2B), the measured levels of Cd were relatively higher in Nariyan and Jinnah Abad, Cr in Sheik ul Bandi, and Ni in Jangi Syedan and Kaghan Colony. Comparatively higher concentration of Pb was noted in the milk samples collected from different shops located in Supply, Malik Pura, and Nawan Sher areas, while Zn concentration was relatively higher in the samples collected from Sheik ul Bandi, Bilal Town, and Damtor.

### 3.2. Comparative Appraisal of Potentially Toxic Metals in Buffalo Milk 

**Figure 3:**
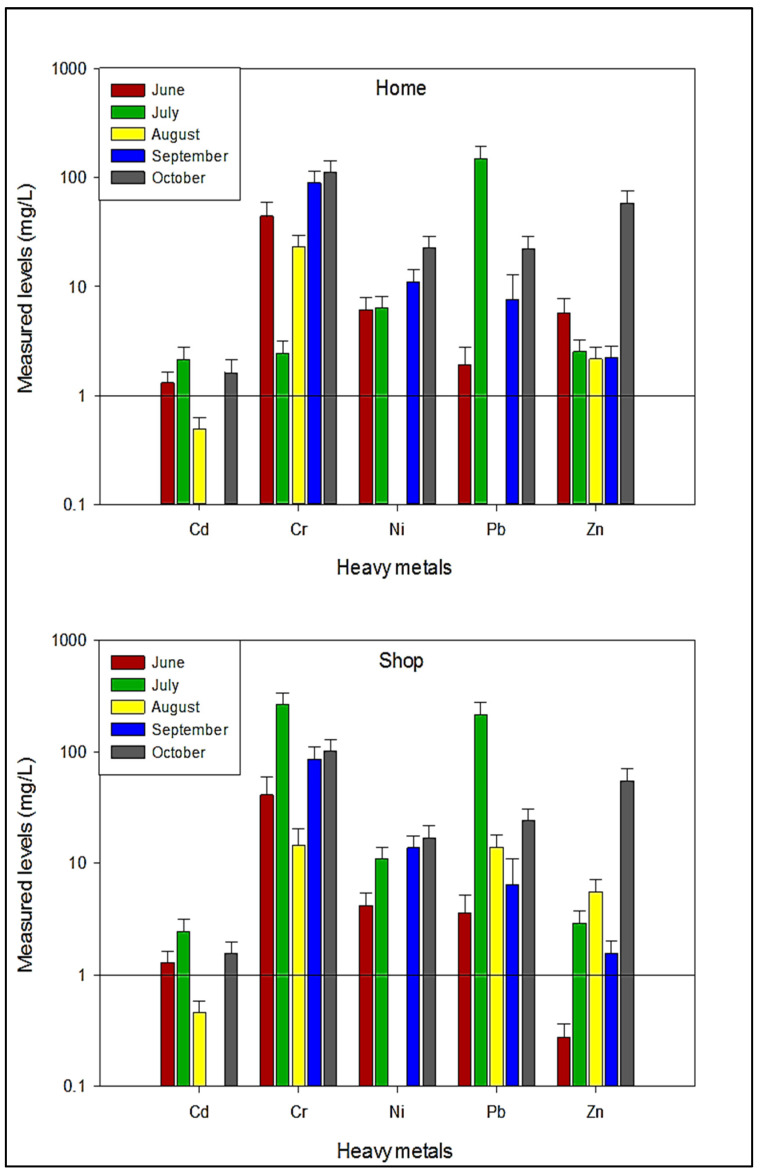
Comparative assessment of average levels of potentially toxic metals in buffalo milk.

Comparative evaluation of the metals quantified in buffalo milk samples collected in the present study from Abbottabad during various sampling months is illustrated in Figure 3. The average concentration of Cd in buffalo milk collected from homes of different localities varied from 0.488 ± 0.135 to 2.145 ± 0.595 mg/L. The milk samples collected in July depicted a relatively higher concentration of Cd, followed by October and June, whereas the lowest Cd level was found in August. Nonetheless, it was below the detection limit in September. Likewise, in the milk samples collected from shops, elevated Cd level was observed in July (2.446 ± 0.678 mg/L), followed by October (1.538 ± 0.488 mg/L) and June (1.281 ± 0.348 mg/L). However, it was not detected in the milk samples collected from shops of all localities in September. The measured levels of Cd in buffalo milk collected from homes and shops of different localities in Abbottabad were relatively higher than the previously reported levels in the same type of milk from Italy [48], Egypt [49,50], Azerbaijan [51], and Pakistan [52]. However, in the present study, the average concentration of Cd was less than the reported levels in buffalo milk from Iran [53,54,55]. 

Exposure to Cd is associated with a variety of health problems, particularly when it is accumulated in the body beyond the threshold level; it may cause anosmia, heart failure, tumors, vomiting, diarrhea, lung damage, fragile bones, cerebrovascular infarction, emphysema, osteoporosis, eye cataract development, proteinuria, etc. [55,56,57]. It also causes various types of cancer and even death [58].

The average concentration of Cr in buffalo milk collected from homes ranged from 111.0 ± 32.04 to 2.446 ± 0.678 mg/L (Figure 3). The highest concentration of Cr was found in October, followed by September, and June, whereas the lowest level was in July. In milk samples collected from shops, Cr was maximum in July (264.4 ± 73.32 mg/L), followed by October and September (101.3 ± 28.09, and 85.78 ± 23.79 mg/L, respectively), while the lowest concentration was noted in August (14.39 ± 5.876 mg/L). Measured levels of Cr in buffalo milk collected from homes and shops of Abbottabad were relatively higher than those reported previously from Italy [48], Egypt [50], and Pakistan [52]. The concentration of Cr in human body exceeding 0.003 mg/kg may cause skin rashes, respiratory problems, kidney, stomach, and lung damage, ulcers, and reduces immunity [59]. In addition, it is also involved in nose irritation, asthma, breathing problems/cough, allergy, redness/swelling, liver damage, and nerve tissue damage [57]. 

The mean concentration of Ni in buffalo milk collected from homes ranged from 22.55 ± 6.256 to 6.388 ± 1.772 mg/L. The highest concentration was recorded in October (22.55 ± 6.256 mg/L), followed by September (11.11 ± 3.081 mg/L), and July (6.388 ± 1.772 mg/L). As illustrated in Figure 3, buffalo milk collected in October contained the highest concentration of Ni in October (16.93 ± 4.696 mg/L), followed by September (13.73 ± 3.809 mg/L), and July (10.91 ± 3.025 mg/L), while Ni was below the detection limit in August. Comparatively higher levels of Ni in the present study were noted than the previously reported levels in Italy [48], Pakistan [52], and Egypt [50]. The permissible limit of Ni in milk is 3–7 mg/day [60], and its concentration exceeding the limit may damage cell structure/DNA [61], and it can cause asthma, headache, nausea, nasal cavity cancer, lung cancer [62], dermatitis, fibrosis [63], heart problems, skin rashes, headache, dizziness, and fatigue [15].

The mean concentration of Pb in buffalo milk collected from homes ranged from 150.1 ± 41.63 to 7.564 ± 5.348 mg/L (Figure 3). The highest concentration of Pb was determined in the samples collected in July (150.1 ± 41.63 mg/L), followed by October (22.33 ± 6.469 mg/L) and September (7.564 ± 5.348 mg/L), while it was below the detection limit in August. In the milk samples of shops, the maximum concentration of Pb was found in July (216.9 ± 0.810 mg/L), followed by October (24.11 ± 6.687 mg/L) and August (14.00 ± 4.041 mg/L), and the lowest was observed in June (3.600 ± 1.610 mg/L). The measured levels of Pb in the present study were comparatively higher than those reported earlier in Italy [48], Egypt [49,50], Azerbaijan [51], and Iran [53,54]. Lead can affect all organs/systems in the human body, especially the central nervous system [64] and may cause death by interfering with the cardiovascular system [65]. In food, a high concentration of Pb can cause behavioral abnormalities, inhibit hemoglobin synthesis, reduce memory, and may cause reproductive failure and neurological disorders [66,67].

As shown in Figure 3, the highest concentration of Zn was recorded in the milk samples collected from homes during October (58.60 ± 16.92 mg/L), followed by June and July (5.657 ± 2.138 and 2.518 ± 0.698 mg/L, respectively), whereas the lowest level was found in August (2.158 ± 0.598 mg/L). In the milk samples of shops, Zn level was maximum in October (54.67 ± 15.16 mg/L), followed by August (5.523 ± 1.532 mg/L) and July (2.921 ± 0.801 mg/L). Interestingly, measured levels of Zn in homes and shops samples collected, particularly in the month of October, were higher than the standard level, which is 5.0 mg/kg for buffalo milk [68]. In addition, measured levels of Zn in buffalo milk collected from homes and shops were relatively higher than the reported levels from Italy [48], West Bengal [69], Egypt [50], and India [70,71]. A higher concentration of Zn in milk may cause various disorders such as kidney and liver failure, reduction in blood lipoprotein [7], gastrointestinal distress, dizziness, and nausea [72]. Overall, the study revealed diverse variations of the metal contents in the milk samples; such variation may be attributed to the contamination and anthropogenic influences of the environment and food chain. 

### 3.3. Correlations Analysis 

A correlation coefficient matrix related to the metals’ concentrations in buffalo milk collected in the current study is shown in Table 2. In the milk samples collected from homes, highly significant positive associations (*p* ≤ 0.05) were noted between Ni and Zn (*r* = 0.884), which were followed by strong positive relationships between Ni-Cr, Pb-Cd, and Zn-Cr. However, Cr depicted negative correlations with Cd and Pb. Conversely, in the milk samples collected from shops, Cr showed a highly significant positive relationship (*p* ≤ 0.05) with Pb and Cd but negative with Zn. Likewise, the correlation analysis was also carried out on the basis of the metals’ concentrations in the buffalo milk collected in different months from various locations (Figure S1—Supplementary Material). On a comparative basis, home milk samples (Figure S1A) collected in August and June showed highly significant positive correlations (*p* ≤ 0.01) with those collected in September (*r* = 1.000 and 0.992, respectively), followed by June with August and October (*r* = 1.000 and 0.912, respectively) with a significant difference (*p* ≤ 0.05). However, there were negative associations among the samples collected in July with September and October. As depicted in Figure S1B, buffalo milk collected in June from various shops located in the study area exhibited a highly significant positive relationship (*p* ≤ 0.01) with those collected in September (*r* = 0.998), followed by July and August (*r* = 0.947) and June and July (*r* = 0.738).

### 3.4. Multivariate Analysis

**Figure 4:**
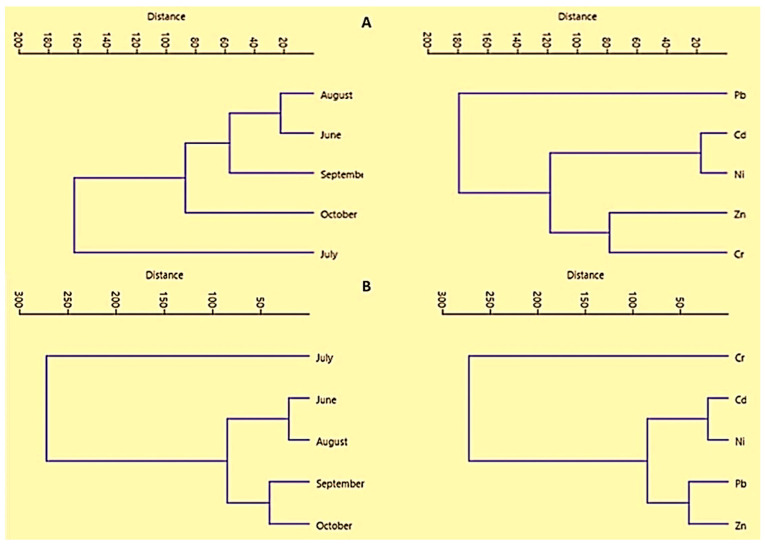
Cluster analysis for the concentrations of potentially toxic metals in buffalo milk: (**A**) home samples, (**B**) shop samples.

To recognize the mutual associations and possible sources of the metals contamination in buffalo milk samples collected from homes and shops, multivariate statistical approaches viz. cluster analysis (CA) and principal component analysis (PCA) were also applied. As demonstrated in Figure 3A, the concentrations of trace metals in home milk samples were categorized into two main clusters, which were further divided into four sub-groups. The milk samples collected in June and August showed an almost similar grouping of the metals, whereas those collected in July showed an entirely different pattern. In addition, the samples collected in September and October also exhibited diverse grouping compared to other months. Likewise, the measured levels of the metals in home milk also depicted different clustering patterns (Figure 4A), and they were classified into three main groups: Zn and Cr (in the first cluster), Ni and Cd (second cluster), while Pb was in a separate group. Cluster analysis of the metals in buffalo milk samples collected from shops (Figure 4B) revealed that in September–October and June–August, concentrations of the metals exhibited comparable associations. However, the distribution of trace metals in the milk samples collected in July exhibited a different trend. Similarly, Pb and Zn were closely associated in a mutual cluster during different months. Likewise, Cd and Ni were placed in the second cluster, but Cr showed a diverse pattern.

The metal levels in buffalo milk samples collected from homes were categorized into two principal components (PC1 and PC2 with variances of 75.57% and 29.42%, respectively) as shown in Figure S2. In PCI, Cr, Zn, and Ni depicted close associations based on loading values of 99.20, 89.20, and 86.00%, respectively. However, Cd and Pb were prevailing in PC2 with 78.70% and 60.90% contributions.

Likewise, the trace metal contents in the milk samples collected from shops were also distributed into two main components; the variance of PC1 was 62.35% and that of PC2 was 37.64%. In the case of PC 1, Pb showed the highest loading value (99.90%), followed by Cd (99.70%) and Cr (99.20%). However, Ni and Zn were closely correlated in PC 2 with contributions of 98.60% and 94.10%, respectively. The grouping of trace metals in two main groups indicates that in shop milk, the concentrations of these metals (Cd, Cr, Pb), and (Ni, Zn) were strongly correlated, and their sources of contamination in the buffalo milk are the same in the study area.

The grouping pattern and sources of the metals appraised by CA and PCA revealed that the metals sharing the mutual group may have common sources of contamination, while the metals showing different trends might have other sources of contamination in the milk collected from homes and shops. For instance, Cd is one of the major eco-toxic metals that occur in the forms of oxides, carbonates, and sulfides (monteponite, octavite, greenockite, respectively), and as minerals in the soil [73]. Yellow paints used for marking roads, chrome plating on different parts of vehicles, different coolants used in engines and air conditioners, and catalytic converters are various anthropogenic sources of Cr contamination of the food chain including milk [74]. Pb is the most abundant among toxic metals [75]. Likewise, different minerals including smithsonite, zincite, sphalerite, mica, and magnetite are common sources of Zn in the soil [76]. However, Zn concentration in food may also enhance due to coal mining, processing in steel industries, and burning of waste materials. In addition, different compounds of Zn are also used as antioxidants, detergents, and brakes for automobiles [77]. Anthropogenic activities such as the extensive use of chemical fertilizers containing micronutrients are another important source of Zn [78]. In addition, the use of phosphate fertilizers containing traces of Cd and Pb as impurities may result in the accumulation of these metals in soil and crops, from where they enter animal bodies [79]. Likewise, different types of fungicides, pesticides, composts, sludge, and livestock manures may also result in the metals’ contamination in the soil and food chain [80,81,82]. Furthermore, mining and smelting of the metal ores (Cr, Pb, and Zn ores) and their processing in industries also triggers the metals contamination, which may cause health risk to ecosystems and humans [82].

### 3.5. Health Risk Assessment

Health risks to the consumers associated with the toxic trace metal contamination in buffalo milk was calculated by considering estimated daily intake (EDI), health risk index (HRI), target hazard quotient (THQ), hazard index (HI), and target cancer risk (TCR). The EDI of the trace metals in adults and children (month-wise) due to the consumption of buffalo milk supplied from local cattle farms (in homes) located in different sites of Abbottabad is mentioned in Table 3. On a comparative scale, the intake of the metals was maximum in October, followed by July and September in both adults and children. 

**Figure 5:**
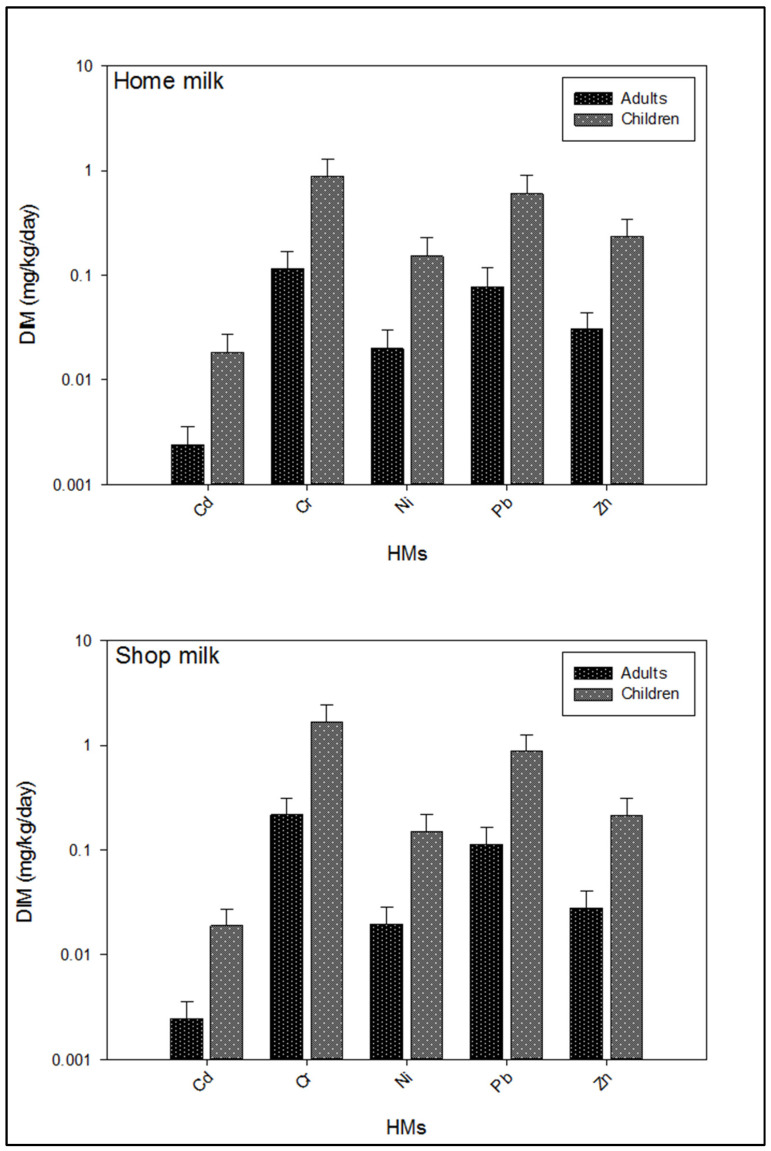
Comparison of estimated daily intake (mg/kg/day) of potentially toxic metals via consumption of buffalo milk.

As shown in Figure 5, Cr was the most ingested among all studied metals with the highest mean intake in adults and children (0.116 and 0.893 mg/kg/day, respectively), followed by Pb and Zn, while the lowest intake was estimated for Cd. In the shop milk (Table 3), the highest intake of metals was estimated in July, followed by October and September both in adults and children. However, the average intake of Cr was highest in adults (0.217 mg/kg/day) and children (1.672 mg/kg/day), followed by Pb and Zn (Figure 5).

**Figure 6:**
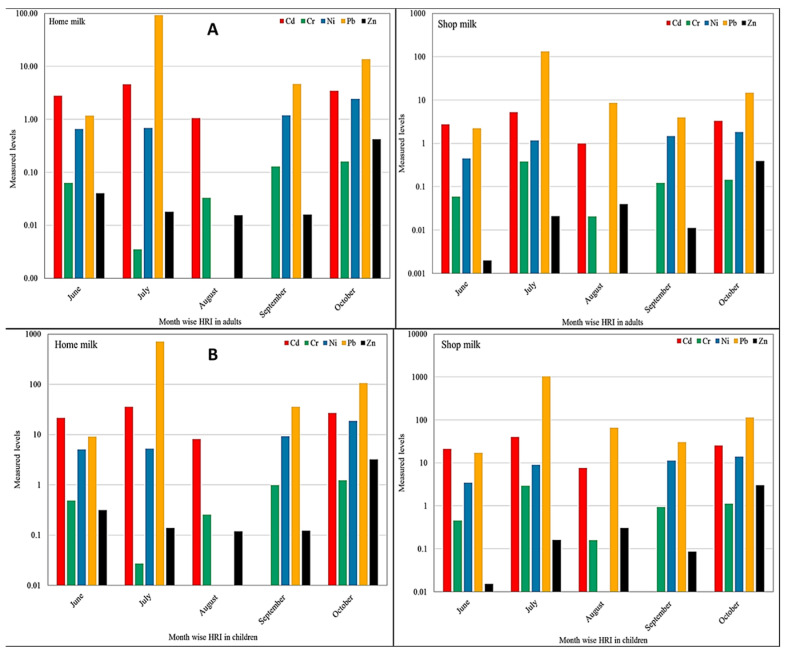
Health risk index of potentially toxic metals in (**A**) adults, (**B**) children.

The health risk index (HRI) calculated for adults and children due to intake of buffalo milk collected from homes and shops is presented in Figure 6. As reported earlier [34], the samples showing HRI values less than one (˂1) are considered safe for human consumption, while those with HRI >1 are deemed harmful. In the milk samples collected from homes and shops, the HRI values of Cr and Zn were ˂1 for adults in all months; it indicated that these metals were within safe limits and posed no considerable harmful effect on the consumers. However, the HRI values of Cd and Pb was >1 in almost all months, indicating that long-time exposure to these metals in consumers via consumption of the milk could be harmful and may impose adverse health effects. In the milk samples collected from homes, the HRI values of all the metals were >1 in October. In June and July, Cd, Ni, and Pb depicted HRI >1, while Zn was within the safe limit, except in October. Almost similar trends were observed for the calculated HRI values in the shop milk. The milk samples collected from shops were relatively more contaminated than the home-based milk samples. This might be due to the fact that in case of the shops, the milk is transported from different areas, and it is stored in metallic containers coated with paints that may result in the metals contamination. In addition, the milk is usually kept in open containers in the shops, which are located along the roadsides; therefore, they are more exposed to the metal’s contamination from the air and automobiles emission as well as nearby industrial emissions. 

**Figure 7:**
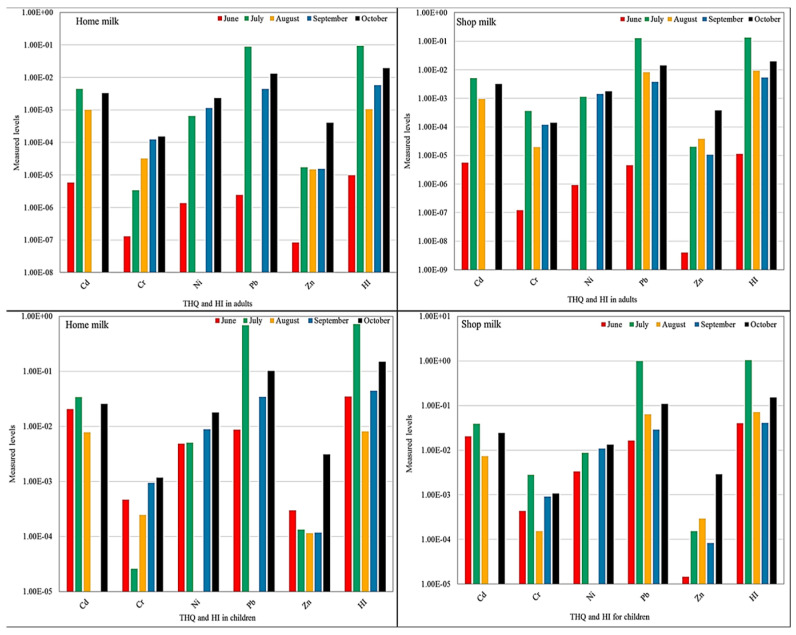
Target hazard quotient and health index of potentially toxic metals for adults and children.

As reported by the United States Environmental Protection Agency [45], the health protection standard of lifetime non-carcinogenic risk in terms of THQ and HI is 1. The calculated values of THQ for the metals in buffalo milk collected from homes and shops were found to be within the safe limit for adults and children (Figure 7). Therefore, the ingestion of buffalo milk by the inhabitants of Abbottabad city was considered safe, and no significant non-carcinogenic risk was associated with it. In addition, the HI values of all metals were also ˂1.0 and were within the safe limit set by [45].

**Figure 8:**
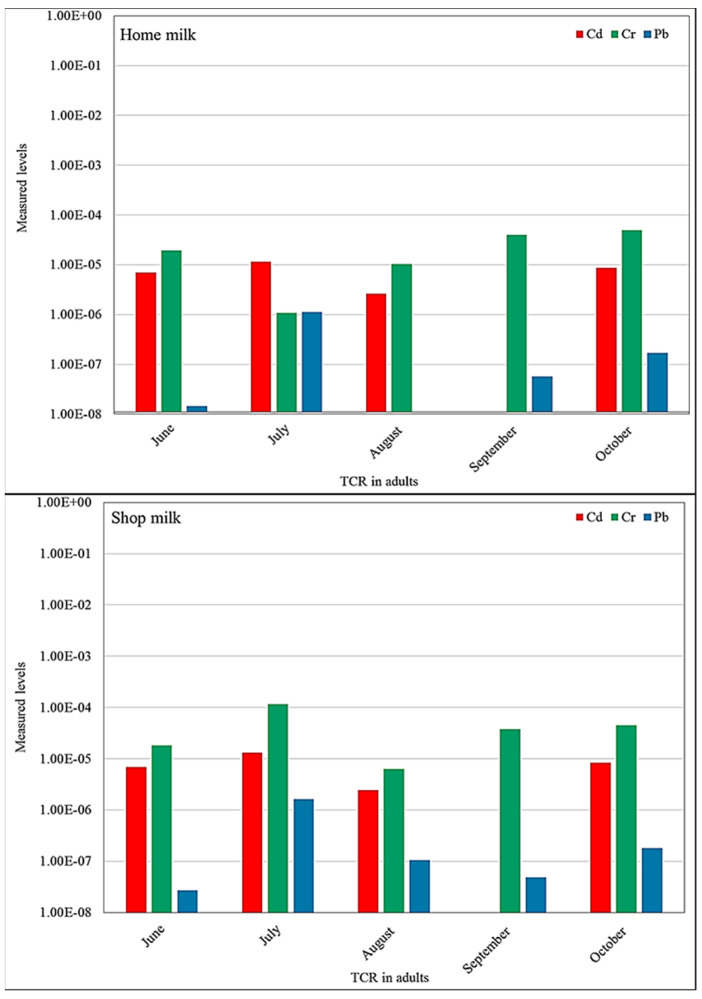
Target cancer risk of potentially toxic metals for adults.

The cancer risk due to the intake of toxic metals by the consumers was estimated for carcinogenic metals viz. Cd, Cr, and Pb. According to [45], acceptable levels of TCR for the metals in food materials ranged from 1 × 10^−4^ to 1 × 10^−6^. As shown in Figure 8, the estimated levels of TCR for Cd, Cr and Pb in buffalo milk collected from homes and shops of different localities of Abbottabad city were within the safe limit in adults. However, the TCR values for Cd in September, and that of Pb in August, October, September, and June were comparatively higher and alarming in the home and shop samples.

## 4. Conclusions

Five potentially toxic metals were quantified in composite samples of buffalo milk. These samples were collected for five consecutive months (June 2021 to October 2021) from homes and shops located at different sites (*n* = 27) in Abbottabad City, Pakistan. The mean concentration of Cr was relatively higher in the milk samples collected from homes and shops, followed by Pb, Zn, Ni, and Cd metals. Overall, the average concentration of Cd, Cr, Ni, Pb, and Zn was relatively higher than most of the previously reported levels, and specifically, the milk samples collected from shops depicted relatively higher levels of the metal’s contamination compared to the home-based milk samples. The concentrations of the metals in home and shop milk samples were relatively higher in October and July, respectively. The univariate and multivariate analysis established strong associations between the metals that were sharing the common sources of contamination. PCA and CA revealed that anthropogenic activities were the major contributors to the metal’s contamination in the milk. Both non-carcinogenic and carcinogenic health risk indices exhibited an insignificant adverse health effect on the consumers due to the metal’s concentration in buffalo milk. However, detailed studies should be conducted, specifically focused on continuous monitoring of the metals and other toxins in various types of fresh and preserved milk throughout the year. In addition, regular monitoring of livestock feed and drinking water quality as well as milk processing and transportation should be strictly ensured.

## Figures and Tables

**Table 1:** Concentrations of potentially toxic metals in buffalo milk collected from homes and shops.

Month	Sampling Sites	Cd		Cr		Ni		Pb		Zn	
		Home	Shop	Home	Shop	Home	Shop	Home	Shop	Home	Shop
June	Mirpur	0.900	1.500	BDL	BDL	20.00	2.050	2.500	13.650	0.200	0.600
	Damtoor	0.550	1.000	BDL	BDL	9.050	6.250	BDL	0.100	0.300	0.450
	Civil Officer Colony	1.350	0.800	63.55	BDL	6.150	3.550	BDL	BDL	0.300	0.100
	Bilal Town	1.200	1.400	BDL	BDL	1.050	9.400	BDL	BDL	0.200	0.100
	Malik Pura	1.550	1.300	82.85	BDL	5.550	5.400	BDL	BDL	0.100	0.100
	Kehal	1.050	1.700	12.90	BDL	0.450	2.550	BDL	1.500	BDL	BDL
	Sheik ul Bandi	1.200	0.900	BDL	BDL	BDL	2.450	2.200	2.250	BDL	BDL
	Nawan Sher	1.050	0.950	BDL	9.250	BDL	1.550	BDL	BDL	BDL	BDL
	Supply	1.150	1.250	78.95	101.6	5.700	4.150	BDL	BDL	BDL	0.050
	Naryian	1.200	1.300	18.55	BDL	8.950	5.000	0.750	BDL	BDL	0.200
	Jangi Syedan	2.100	1.500	26.95	27.95	2.550	BDL	0.550	BDL	0.150	0.050
	Jinnah Abad	1.200	1.600	38.05	11.35	BDL	4.050	3.600	0.500	BDL	0.100
	Kaghan Colony	1.550	1.450	38.35	54.75	2.400	3.750	BDL	BDL	38.35	1.000
	Mean	1.235	1.281	45.02	40.97	6.185	4.179	1.920	3.600	5.657	0.275
	SE	0.330	0.355	15.92	18.32	1.865	1.206	0.859	1.610	2.138	0.087
July	Mirpur	0.750	0.950	102.8	90.95	BDL	21.20	92.40	BDL	4.000	1.250
	Damtoor	0.950	1.100	40.00	48.35	1.350	6.800	162.8	234.1	4.100	4.450
	Civil Officer Colony	0.800	1.400	94.30	21.60	5.800	1.150	BDL	8.900	3.600	2.700
	Bilal Town	1.750	2.100	BDL	BDL	4.850	8.000	39.55	BDL	3.250	3.150
	Malik Pura	1.900	2.200	BDL	BDL	5.350	BDL	BDL	293.1	1.300	6.550
	Kehal	2.150	2.400	BDL	BDL	10.75	15.85	BDL	BDL	1.750	3.000
	Sheik ul Bandi	2.550	2.900	BDL	896.5	7.400	12.45	214.7	270.2	2.950	3.350
	Nawan Sher	2.950	2.900	BDL	BDL	9.950		314.7	312.1	3.400	3.000
	Supply	3.000	3.250	BDL	BDL	BDL	BDL	BDL	346.4	2.350	3.050
	Naryian	3.250	3.350	BDL	BDL	5.650	BDL	76.50		2.650	1.550
	Jangi Syedan	3.100	3.550	BDL	BDL	BDL	BDL	BDL	90.55	1.300	1.850
	Jinnah Abad	3.350	3.250	BDL	BDL	BDL	BDL	BDL		1.050	2.250
	Kaghan Colony	3.450	BDL	BDL	BDL	BDL	BDL	BDL	180.3	3.500	2.450
	Mean	2.145	2.446	79.03	264.4	6.388	10.91	150.1	216.9	2.518	2.921
	SE	0.595	0.678	21.92	73.32	1.772	3.025	41.63	60.17	0.698	0.810
August	Mirpur	0.250	0.600	45.10	9.950	BDL	BDL	BDL	BDL	3.500	3.850
	Damtoor	0.500	0.250	19.85	7.100	BDL	BDL	BDL	BDL	3.450	8.600
	Civil Officer Colony	0.600	0.550	2.750	18.10	BDL	BDL	BDL	BDL	2.400	3.000
	Bilal Town	0.450	0.550	41.55	BDL	BDL	BDL	BDL	BDL	3.000	11.650
	Malik Pura	0.700	0.500	27.40	28.75	BDL	BDL	BDL	BDL	1.650	2.000
	Kehal	0.550	0.400	BDL	8.500	BDL	BDL	BDL	BDL	1.650	5.400
	Sheik ul Bandi	0.400	0.700	2.450	BDL	BDL	BDL	BDL	BDL	2.600	3.650
	Nawan Sher	0.200	0.150	BDL	BDL	BDL	BDL	BDL	BDL	1.950	2.550
	Supply	0.500	0.500	BDL	BDL	BDL	BDL	BDL	BDL	1.700	7.800
	Naryian	0.500	0.400	BDL	2.350	BDL	BDL	BDL	BDL	1.800	2.000
	Jangi Syedan	0.400	0.200	BDL	26.00	BDL	BDL	BDL	BDL	1.900	8.600
	Jinnah Abad	0.600	0.550	BDL	BDL	BDL	BDL	BDL	BDL	1.450	7.400
	Kaghan Colony	0.700	0.600	BDL	BDL	BDL	BDL	BDL	14.00	1.000	5.300
	Mean	0.488	0.458	23.183	14.393				14.00	2.158	5.523
	SE	0.135	0.127	6.430	5.876				4.041	0.598	1.532
September	Mirpur	BDL	BDL	81.25	65.90	5.100	27.20	10.40	7.700	2.450	0.850
	Damtoor	BDL	BDL	75.55	71.75	10.15	9.800	7.550	5.300	1.750	0.400
	Civil Officer Colony	BDL	BDL	63.15	65.65	10.25	12.55	7.800	6.750	4.150	2.800
	Bilal Town	BDL	BDL	86.95	69.65	11.50	9.300	11.05	5.250	2.450	2.400
	Malik Pura	BDL	BDL	81.60	75.80	4.900	10.50	10.45	9.250	2.550	3.000
	Kehal	BDL	BDL	94.55	80.55	8.350	11.95	5.150	BDL	4.650	0.950
	Sheik ul Bandi	BDL	BDL	90.65	82.75	12.45	6.050	5.350	8.300	0.900	1.850
	Nawan Sher	BDL	BDL	94.40	104.8	6.200	12.90	8.950	6.550	0.900	0.600
	Supply	BDL	BDL	90.70	90.05	5.600	7.300	4.350	3.950	0.850	1.550
	Naryian	BDL	BDL	94.40	95.95	12.75	12.50	2.200	3.450	0.350	0.700
	Jangi Syedan	BDL	BDL	99.65	103.1	23.30	19.95	BDL	6.050	1.600	0.950
	Jinnah Abad	BDL	BDL	93.90	100.8	15.20	21.80	BDL	BDL	1.600	1.150
	Kaghan Colony	BDL	BDL	118.8	108.4	18.65	16.75	9.950	7.900	4.800	3.050
	Mean			89.65	85.78	11.11	13.73	7.564	6.405	2.231	1.558
	SE			24.87	23.79	3.081	3.809	5.348	4.529	0.619	0.432
October	Mirpur	0.950	2.050	49.35	160.8	18.35	6.80	18.35	11.55	55.50	26.40
	Damtoor	0.150	0.100	65.30	89.00	17.90	9.60	17.90	15.65	72.70	65.95
	Civil Officer Colony	BDL	1.750	142.1	127.7	14.90	8.30	14.90	22.50	63.05	59.55
	Bilal Town	BDL	BDL	191.0	130.8	14.50	12.50	14.50	17.60	65.85	65.50
	Malik Pura	1.250	0.850	214.3	147.5	27.70	16.95	27.70	18.30	76.00	66.00
	Kehal	0.600	0.950	161.5	108.3	29.20	17.70	29.20	31.55	78.40	46.00
	Sheik ul Bandi	1.850	1.400	74.20	143.0	16.40	18.90	16.40	26.30	57.10	59.25
	Nawan Sher	3.100	1.500	48.45	85.70	18.55	24.30	18.55	25.25	47.05	67.55
	Supply	1.450	1.850	86.85	2.55	21.20	19.85	21.20	31.85	45.45	44.70
	Naryian	2.750	3.300	160.4	13.80	23.30	23.40	23.30	26.45	30.95	56.65
	Jangi Syedan	2.300	1.650	75.00	69.55	24.95	16.15	24.95	24.65	69.75	55.40
	Jinnah Abad	1.750	1.950	BDL	BDL	43.15	19.90	43.15	22.30	BDL	50.55
	Kaghan Colony	1.650	1.100	63.55	116.7	23.15	25.75	33.15	39.50	41.45	47.20
	Mean	1.618	1.538	111.0	101.3	22.56	16.93	22.34	24.11	58.60	54.67
	SE	0.488	0.444	32.04	28.09	6.256	4.696	6.469	6.687	16.92	15.16

BDL. Bellow the detection limit.

**Table 2:** Correlation coefficient matrix for the concentrations of potentially toxic metals in buffalo milk.

Sites	Variables	Cd	Cr	Ni	Pb	Zn
Home	Cd	1.000	−0.298	0.236	0.715 *	0.345
	Cr		1.000	0.843 *	−0.536	0.701 *
	Ni			1.000	−0.056	0.884 *
	Pb				1.000	−0.137
	Zn					1.000
Shop	Cd	1.000	0.941 *	0.241	0.834 *	0.054
	Cr		1.000	0.199	0.943 *	−0.005
	Ni			1.000	0.004	0.694
	Pb				1.000	−0.163
	Zn					1.000

* Correlation is significant at *p* < 0.01.

**Table 3:** Month wise EDI (mg/kg/day) of potentially toxic metals via consumption of buffalo milk.

Samples		HMs	June	July	August	September	October
Home milk samples	Adults	Cd	0.003	0.005	0.001	0.000	0.003
		Cr	0.095	0.005	0.050	0.192	0.238
		Ni	0.013	0.014	0.000	0.024	0.048
		Pb	0.004	0.322	0.000	0.016	0.048
		Zn	0.012	0.005	0.005	0.005	0.126
	Children	Cd	0.022	0.035	0.008	0.000	0.027
		Cr	0.731	0.040	0.383	1.479	1.832
		Ni	0.101	0.105	0.000	0.183	0.372
		Pb	0.032	2.477	0.000	0.125	0.368
		Zn	0.093	0.042	0.036	0.037	0.967
Shop milk samples	Adults	Cd	0.003	0.005	0.001	0.000	0.003
		Cr	0.088	0.566	0.031	0.184	0.217
		Ni	0.009	0.023	0.000	0.029	0.036
		Pb	0.008	0.465	0.030	0.014	0.052
		Zn	0.001	0.006	0.012	0.003	0.117
	Children	Cd	0.021	0.040	0.008	0.000	0.025
		Cr	0.676	4.362	0.237	1.415	1.671
		Ni	0.069	0.180	0.000	0.227	0.279
		Pb	0.059	3.579	0.231	0.106	0.398
		Zn	0.005	0.048	0.091	0.026	0.902

## Data Availability

Not applicable.

## Supplementary Materials

Supporting information can be downloaded at: https://www.mdpi.com/article/10.3390/ijerph192214678/s1, Table S1: Optimum analytical conditions for the analysis of selected trace metals on AAS. Figure S1. Correlations between different months based on the concentrations of potentially toxic metals in buffalo milk. (A) Home milk samples, (B) shop milk samples. Figure S2. Principal component analysis for the concentrations of potentially toxic metals in buffalo milk collected from shops.
