# Supplementary material for: Evaluation of Potentially Toxic Trace Metals and Associated Health Risk Assessment in Buffalo Milk
Source: Int J Environ Res Public Health. 2022 Nov 9;19(22):14678. doi: 10.3390/ijerph192214678 (PMC9691157; doi:10.3390/ijerph192214678)
Supplement: Supplementary file 1 [file ijerph-19-14678-s001.zip › ijerph-1944298-supplementary.pdf]

Table S1. Optimum analytical conditions for the analysis of selected trace metals on AAS.

Conditions	Cd	Cr	Ni	Pb	Zn
Wavelength (nm)	228.8	357.9	232.0	217.0	213.9
Slit width (nm)	0.30	0.50	0.15	0.30	0.50
Lamp current (mA)	4.0	5.0	4.0	7.0	4.0
Acetylene flow rate (L/min)	1.8	2.6	1.7	1.8	2.0
Limit of Detection (μg/L)	4	6	6	9	2
Limit of Quantification (μg/L)	13	18	19	28	6

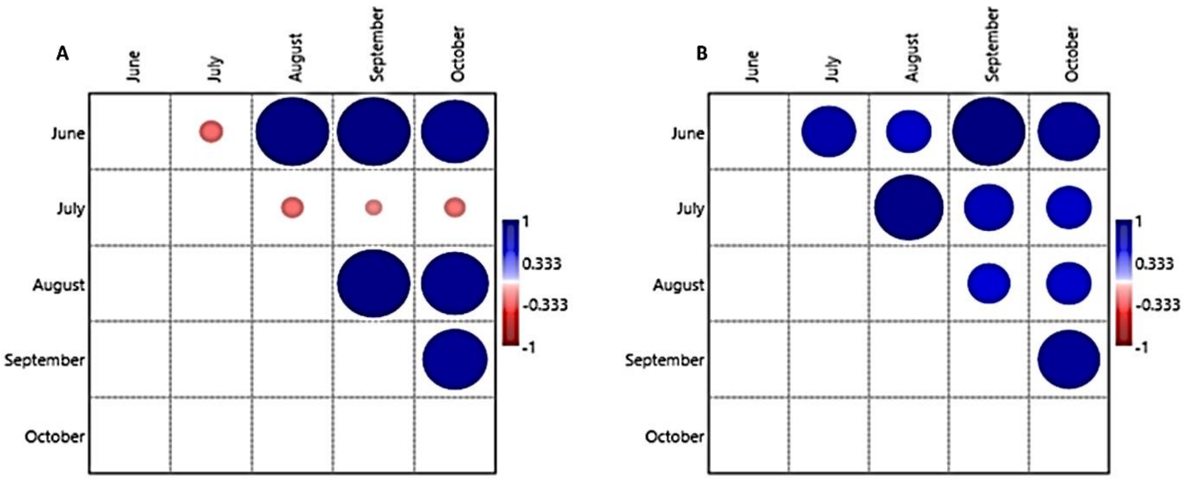

Figure S1. Correlations between different months based on the concentrations of potentially toxic metals in buffalo milk. (A) home milk samples, (B) shop milk samples

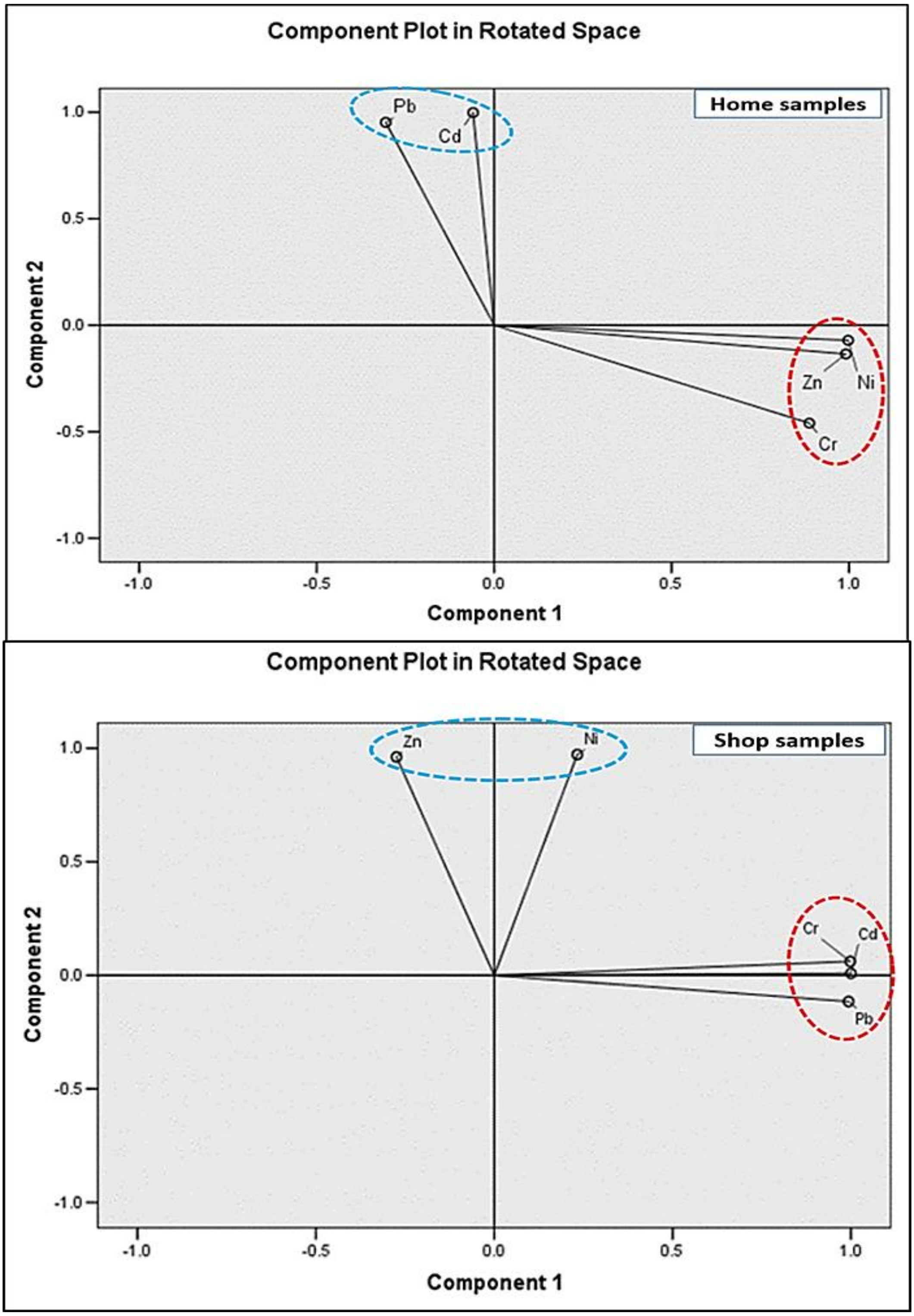

Figure S2. Principal component analysis for the concentrations of potentially toxic metals in buffalo milk collected from homes and shops
